# Microscopic Polyangiitis Following Kawasaki Disease in a Patient With Autoimmune Predisposition

**DOI:** 10.7759/cureus.111020

**Published:** 2026-06-17

**Authors:** Ayako Kamiya, Shoichiro Kanda, Keiichi Takizawa, Yuko Kajiho, Yutaka Harita

**Affiliations:** 1 Pediatrics, The University of Tokyo, Tokyo, JPN

**Keywords:** anca-associated vasculitis, immune priming, kawasaki disease (kd), microscopic polyangiitis, systemic autoimmune disease

## Abstract

Antineutrophil cytoplasmic antibody (ANCA)-associated vasculitis (AAV) is rare in children, and its pathogenesis remains incompletely understood. Current evidence suggests that complex interactions between genetic susceptibility and environmental triggers contribute to disease development. We report a case of pediatric-onset microscopic polyangiitis (MPA) preceded by recurrent cutaneous manifestations and occurring in a patient with a history of Kawasaki disease and a strong family history of autoimmune diseases.

A 14-year-old boy presented with recurrent painful edematous erythema involving the palms, wrists, and dorsum of the feet. Several months later, he developed fever, arthralgia, oral aphthous ulcers, and weight loss. Urinalysis revealed hematuria and proteinuria, and serum myeloperoxidase (MPO)-ANCA levels were markedly elevated. Renal biopsy demonstrated pauci-immune crescentic glomerulonephritis, leading to the diagnosis of MPA. Remission induction therapy with corticosteroids and cyclophosphamide achieved sustained clinical remission without renal dysfunction.

The patient had a history of Kawasaki disease in infancy, more than a decade before the onset of MPA. In addition, his mother had rheumatoid arthritis and myasthenia gravis, and his younger sister had immune thrombocytopenic purpura. This case raises the possibility that prior immune-mediated vascular inflammation, together with autoimmune genetic predisposition, may contribute to the later development of MPO-ANCA-associated vasculitis. Further accumulation of similar cases may help clarify the immunopathogenic relationship between Kawasaki disease and AAV.

## Introduction

Antineutrophil cytoplasmic antibody (ANCA)-associated vasculitis (AAV) is a group of necrotizing vasculitides predominantly affecting small vessels and comprises three major entities: microscopic polyangiitis (MPA), granulomatosis with polyangiitis, and eosinophilic granulomatosis with polyangiitis. AAV primarily occurs in adults, and pediatric-onset cases are relatively rare. Recent epidemiological data estimate the annual incidence of childhood-onset AAV at approximately 3.2 cases per million children, of which MPA accounts for nearly half [1]. In Japan, MPA represents the predominant subtype of pediatric AAV [2].

Renal involvement is common in pediatric AAV and represents a major determinant of long-term outcomes. Previous studies have reported that approximately 10%-50% of pediatric patients with AAV eventually progress to end-stage renal disease (ESRD) [2,3]. In Japan, many cases are detected through abnormalities such as hematuria or proteinuria identified during school urinary screening programs [2,4]. However, nonspecific systemic manifestations, including skin rash, fever, and arthralgia, may precede renal findings in some patients, which can make the diagnosis challenging.

Although the exact pathogenesis of AAV remains incompletely understood, it is considered an autoimmune disease resulting from the interaction between genetic susceptibility and environmental triggers. Genetic association studies have identified several susceptibility loci, particularly within the HLA region [5]. In addition, infections [6], medications [7], and environmental exposures [8] have been proposed as potential triggers of disease onset, suggesting that complex interactions between genetic background and environmental factors contribute to the development of AAV.

Herein, we report a case of pediatric-onset MPA that was preceded by recurrent cutaneous manifestations and was diagnosed by renal biopsy after the detection of hematuria, proteinuria, and markedly elevated myeloperoxidase (MPO)-ANCA levels. This case is of particular interest because the disease was initially suggested by skin manifestations and because the patient had a history of Kawasaki disease and a family history of autoimmune disorders. The coexistence of Kawasaki disease and subsequent MPA has rarely been reported, and the potential immunological relationship between these conditions remains poorly understood. This case may provide insight into how prior immune-mediated vascular inflammation and autoimmune predisposition could contribute to the later development of MPO-ANCA-associated vasculitis. We aimed to describe the clinical course of pediatric-onset MPA following Kawasaki disease and discuss the potential roles of autoimmune predisposition and prior immune-mediated vascular inflammation in disease development.

## Case presentation

A 14-year-old boy presented with a one-year history of recurrent painful edematous erythema with warmth involving the palms, wrists, and dorsum of the feet. The lesions typically resolved within 2-3 days while migrating to other sites, and each episode completely resolved within approximately one week. Similar episodes recurred four times over a one-year period. During the period of recurrent cutaneous manifestations, the patient was evaluated by the dermatology department. A skin biopsy was performed and demonstrated inflammatory changes involving the deep dermis, subcutaneous tissue, and superficial fascia. Dense neutrophil-predominant inflammatory cell infiltration with admixed eosinophils, lymphocytes, and plasma cells was observed, accompanied by nuclear debris and secondary vasculitic changes. Mild perivascular inflammatory cell infiltration was also present in the superficial dermis. No granulomas, fat necrosis, microorganisms, or definitive features of primary vasculitis were identified. These findings were considered nonspecific and were insufficient to establish a definitive diagnosis. Although the biopsy findings were nonspecific, these recurrent cutaneous manifestations may have represented an early inflammatory phase preceding the development of systemic vasculitis. Serological testing for autoimmune diseases, including anti-double-stranded DNA antibody, anti-SS-A/SS-B antibody, anti-cyclic citrullinated peptide antibody, and anti-Jo-1 antibody, was negative.

Three months later, he developed recurrent fever (>38°C), arthralgia involving large joints, and oral aphthous ulcers, accompanied by an unintentional weight loss of approximately 5-6 kg. Based on the recurrent fever, arthralgia, oral aphthous ulcers, and skin lesions, Behçet disease and other inflammatory or vasculitic disorders were initially considered. Pathergy testing, ophthalmologic examination, and HLA-B51 testing were not performed. However, Behçet disease became less likely because genital ulcers were absent, and subsequent evaluation revealed hematuria, proteinuria, and markedly elevated MPO-ANCA levels, which suggested ANCA-associated vasculitis. He was referred to our department for further evaluation. There was no history of drug exposure or infection known to induce ANCA-associated vasculitis.

His past medical history was notable for Kawasaki disease in infancy. His family history was significant for autoimmune diseases: his mother had rheumatoid arthritis and myasthenia gravis, and his younger sister had immune thrombocytopenic purpura.

On admission, his blood pressure was 102/64 mmHg. His height was 175 cm (91st percentile), and his weight was 63.4 kg (73rd percentile). Physical examination was largely unremarkable except for small aphthous ulcers in the oral cavity. Laboratory testing revealed mild anemia (hemoglobin: 10.1 g/dL) and elevated inflammatory markers (C-reactive protein: 1.37 mg/dL, erythrocyte sedimentation rate: 62 mm/h). Serum creatinine was 0.82 mg/dL, corresponding to an estimated glomerular filtration rate (eGFR) of 99.7 mL/min/1.73 m², indicating preserved renal function. Immunological testing showed markedly elevated MPO-ANCA levels (516 IU/mL, reference range: 0-5 IU/mL), whereas PR3-ANCA was negative. Urinalysis demonstrated hematuria (3+) and proteinuria (1+), with red blood cell casts in the urinary sediment. Quantitative proteinuria was mild, with a peak urinary protein-to-creatinine ratio of 304.7 mg/gCr (reference value: <150 mg/gCr).

**Figure 1:**
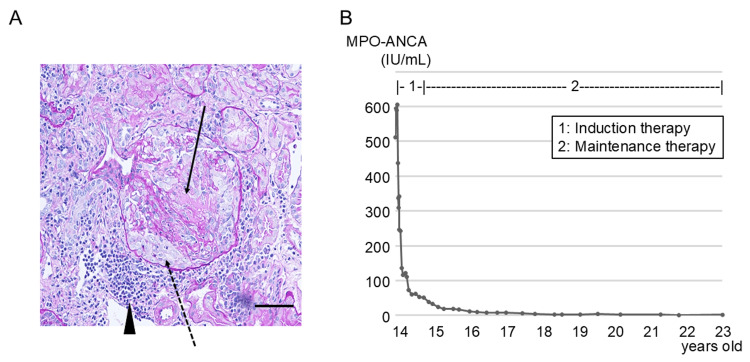
Renal biopsy findings and clinical course A: PAS staining demonstrating a representative glomerulus with cellular crescent formation and fibrinoid necrosis, consistent with crescentic glomerulonephritis. Fibrinoid necrosis (solid arrow), cellular crescent formation (dashed arrow), and inflammatory cell infiltration in the periglomerular area (arrowheads) are shown. Original magnification ×400. Scale bar = 50 μm. B: The patient received remission induction therapy consisting of intravenous methylprednisolone pulse therapy (three courses) followed by six courses of intravenous cyclophosphamide pulse therapy during the first six months after diagnosis. Oral prednisolone was initiated at 45 mg/day and gradually tapered to 10 mg/day over approximately seven months, and maintenance therapy with azathioprine was subsequently administered. MPO-ANCA levels gradually decreased and remained within the normal range without relapse during long-term follow-up. Renal function remained preserved throughout follow-up. Proteinuria was mild (peak urinary protein-to-creatinine ratio: 304.7 mg/gCr, reference value: <150 mg/gCr) and resolved after remission induction therapy. PAS: periodic acid-Schiff, MPO-ANCA: myeloperoxidase-antineutrophil cytoplasmic antibody

A renal biopsy specimen contained 57 glomeruli, 8 (14%) of which showed cellular crescents and 2 demonstrated fibrinoid necrosis (Figure 1A). Prominent inflammatory cell infiltration was observed in the periglomerular area. None showed global glomerulosclerosis. Immunofluorescence microscopy revealed no deposition of immunoglobulins or complement. These findings were consistent with pauci-immune crescentic glomerulonephritis. Although immune complex-mediated glomerulonephritis, including lupus nephritis and IgA vasculitis nephritis, was considered in the differential diagnosis, the absence of immunoglobulin and complement deposition together with MPO-ANCA positivity supported the diagnosis of ANCA-associated vasculitis, leading to the diagnosis of MPA.

Remission induction therapy consisting of intravenous methylprednisolone pulse therapy followed by intravenous cyclophosphamide was initiated. MPO-ANCA levels gradually decreased, and urinary abnormalities resolved. Maintenance therapy with azathioprine and prednisolone was subsequently introduced. During long-term follow-up, MPO-ANCA levels normalized, and the patient has remained in remission without relapse or renal dysfunction up to the age of 23 years (Figure 1B). A summary of the clinical course, major findings, treatment, and outcome is provided in Table 1.

**Table 1:** Clinical course and major findings MPO-ANCA: myeloperoxidase-antineutrophil cytoplasmic antibody, eGFR: estimated glomerular filtration rate, MPA: microscopic polyangiitis

Time point	Clinical findings and management
Infancy	Kawasaki disease treated medically; complete clinical recovery
Family history	Mother: rheumatoid arthritis and myasthenia gravis, younger sister: immune thrombocytopenic purpura
Age 13-14 years	Recurrent painful edematous erythema with warmth involving the palms, wrists, and dorsum of the feet; individual lesions resolved within 2-3 days while migrating to other sites; each episode resolved within approximately 1 week; four episodes occurred over 1 year
Age 14 years (3 months before diagnosis)	Recurrent fever (>38°C), arthralgia, oral aphthous ulcers, and unintentional weight loss (5-6 kg)
Age 14 years (at diagnosis)	MPO-ANCA 516 IU/mL (reference range: 0-5 IU/mL), hematuria (3+), proteinuria (1+), urinary protein-to-creatinine ratio: 304.7 mg/gCr (reference value: <150 mg/gCr), eGFR: 99.7 mL/min/1.73 m²
Renal biopsy	Pauci-immune crescentic glomerulonephritis; 8/57 glomeruli with cellular crescents and 2/57 with fibrinoid necrosis
Diagnosis	MPA
Remission induction therapy	Intravenous methylprednisolone pulse therapy (3 courses) followed by intravenous cyclophosphamide pulse therapy (6 courses)
Maintenance therapy	Prednisolone tapering and azathioprine
Age 23 years (latest follow-up)	MPO-ANCA normalized; sustained remission without relapse or renal dysfunction

## Discussion

This case describes pediatric-onset MPA that was initially suggested by recurrent cutaneous manifestations. At 14 years of age, the patient repeatedly developed edematous erythema involving the palms, wrists, and dorsum of the feet, followed by systemic symptoms including fever, arthralgia, and weight loss. Cutaneous manifestations of AAV may precede overt renal or systemic disease and are often heterogeneous, ranging from palpable purpura and petechiae to nodules, livedo, ulcers, urticarial or maculopapular eruptions, and oral lesions [9,10]. Therefore, even recurrent nonspecific inflammatory skin lesions, such as those observed in our patient, may provide an important early diagnostic clue when accompanied by systemic symptoms or urinary abnormalities. In the present case, further evaluation prompted by these findings revealed hematuria and proteinuria, and markedly elevated serum MPO-ANCA levels together with renal biopsy findings of pauci-immune crescentic glomerulonephritis led to the diagnosis of MPA.

An important characteristic of this case was the strong family history of autoimmune diseases. His mother had rheumatoid arthritis and myasthenia gravis, and his younger sister had immune thrombocytopenic purpura. Although the precise pathogenesis of AAV remains incompletely understood, the disease is generally considered to arise from interactions between genetic susceptibility and environmental triggers. Genetic association studies have identified several susceptibility loci for AAV, particularly within the HLA region. In Japanese populations, the *HLA-DRB1*09:01-*DQB1*03:03 haplotype has been associated with susceptibility to MPA [11]. Interestingly, this haplotype has also been implicated in other autoimmune diseases [12], including rheumatoid arthritis and myasthenia gravis, suggesting a shared immunogenetic background among these disorders. However, HLA typing and genetic testing were not performed in this patient; therefore, the proposed immunogenetic relationship remains speculative.

Another notable feature of this case was the patient's history of Kawasaki disease in early childhood. Kawasaki disease is an acute systemic vasculitis characterized by dysregulated immune activation. Although a direct relationship between Kawasaki disease and AAV has not been established, the coexistence of these two vasculitic conditions raises the possibility that prior vascular inflammation and immune dysregulation may influence later susceptibility to autoimmune vasculitis. MPO-ANCA has been detected in some patients with Kawasaki disease [13], suggesting that neutrophil-targeted autoimmunity may occur during Kawasaki-related vascular inflammation. Furthermore, experimental studies have demonstrated that *Candida albicans*-derived water-soluble mannoprotein fractions can induce coronary arteritis resembling Kawasaki disease in mice [14]. In this model, MPO-ANCA levels gradually increase, and administration of MPO-ANCA further exacerbates vascular inflammation [15]. Together, these findings support the hypothesis that antigen-driven vascular inflammation may contribute to the priming of neutrophil-directed autoimmunity. In this context, Kawasaki disease may be viewed as a potential immune-priming event preceding the later development of MPO-ANCA-associated vasculitis. Such immune priming implies the possibility that a preceding inflammatory condition may influence subsequent immune responses without necessarily indicating a causal relationship. Consistent with this concept, overlap or sequential occurrence of distinct vasculitic disorders has been described. Reports describing Kawasaki disease preceding ANCA-associated vasculitis are extremely limited. Kawasaki disease has been reported in association with IgA vasculitis, and a cross-phenotype genetic meta-analysis identified a shared susceptibility locus between these conditions, suggesting partially overlapping immunogenetic pathways among pediatric vasculitides [16]. These findings support the possibility that apparently distinct forms of childhood vasculitis may share common mechanisms of immune dysregulation.

Taken together, although a causal relationship cannot be established from a single case, these observations raise the possibility that immune-mediated vascular inflammation in childhood, such as Kawasaki disease, may represent a potential immune-priming event for the later development of MPO-ANCA-associated vasculitis in genetically susceptible individuals.

## Conclusions

In conclusion, we report a rare case of pediatric-onset MPA preceded by Kawasaki disease and occurring in the setting of a strong family history of autoimmune diseases. This case highlights the potential interplay between genetic predisposition and prior immune-mediated vascular inflammation in the pathogenesis of MPO-ANCA-associated vasculitis. Further accumulation of similar cases may help clarify the immunological relationship between Kawasaki disease and later development of AAV.
